# Hypertensive emergencies: a new clinical approach

**DOI:** 10.1186/s40885-015-0027-4

**Published:** 2015-08-13

**Authors:** Alfonso Lagi, Simone Cencetti

**Affiliations:** Emergency & Accident Unit, Ospedale Santa Maria Nuova, ASL 10, Florence, Italy

**Keywords:** Hypertensive emergencies, Malignant hypertension, Accelerated hypertension, Renal crisis

## Abstract

The expression ‘hypertensive urgencies’ includes many diseases. The unifying features of these diseases are a high level of arterial pressure and acute distress of one or more organs. The aim of the review was to define the idea of the ‘acute hypertension’ as a new concept, different from ‘chronic hypertension’. Acute hypertension might be related to ‘organ damage’ because it is the cause, the consequence or an effect of the acute stress. We compounded a narrative review which has included analyses of 373 articles. The structure of the search strategy included a literature search of PubMed, MEDLINE, Cochrane Library and Google Scholar databases. We applied the following inclusion criteria: prospective double-blind randomised controlled trials, experimental animal work studies, case–control studies and recruiting patients representative of the general sick population. In this review, the diseases included in the term ‘hypertensive emergencies’ share ‘acute’ hypertension. This is a new idea that emphasises the suddenly increased arterial pressure, irrespective of the initial arterial pressure and independent of the goals of hypertension control. The ‘hypertensive emergencies’ have been grouped together in three subsets: (1) diseases that result from acute hypertension that is caused by faulty regulation of the peripheral circulation (*acute primary hypertension*), (2) diseases that produce hypertension (*acute secondary hypertension*) and 3) diseases that have hypertension as an effect of the acute stress caused by the principle disease (*acute associated hypertension*). This review highlights a novel idea: acute hypertension is a common sign of different diseases characterised by the sudden surge of arterial pressure, so overwhelming the difference between hypertensive emergencies and urgencies. The judgment of acute hypertension is independent of the initial arterial pressure, normotension or hypertension and is linked with the transient failure of the baroreflex. Hypertensive emergencies are grouped together because all of these diseases require prompt therapy to prevent the negative outcomes of acute hypertension

## Introduction

This work was performed with the aim to make further distinctions between ‘hypertensive emergencies’ and ‘hypertensive urgencies’. The words do not seem appropriate to the clinical importance and are misinterpreted. An increase in arterial pressure alone is not sufficient for grouping these different diseases, which share one sign only. It is difficult to unify diseases with such different clinical features.

In accordance with the literature, we define ‘hypertensive emergencies’ as the sudden and prolonged increase of arterial pressure related to ‘organ damage’ and causing poor outcomes.

With this explanation, we want to go beyond the idea that the hypertension causes ‘organ damage’ or vice versa. We want to show that a temporal relationship exists, but one cannot confirm if hypertension or ‘organ damage’ is the cause or the effect. We say that it is not important whether the disease (organ damage) or the hypertension comes first; the hypertension must be treated because it causes worse outcomes.

In terms of hypertensive emergencies, some items have not been determined: how much the arterial pressure must increase, compared with the initial pressure that is considered normal for the patient if he is normally hypertensive or normotensive (<140/90 mmHg); how sudden the increase in arterial pressure must be or how steep the increase should be; and, finally, the required duration of the hypertension.

To understand the clinical differences of ‘hypertensive emergencies’, it means to take account of the following issues:

- The baroreflex, if appropriate, would not allow the surge of the hypertensive crisis. It (the hypertensive crisis) would be corrected and controlled to lower values regardless of the cause of the hypertension. The baroreflex might be temporarily disrupted, blinded or reset to zero for the period of the crisis, as occurs during physical activity, when hypertension and tachycardia are permitted [1].
- The acute reaction to stress is important. When an acute disease arises, hormones that can increase the arterial pressure are released (ACTH–Cortisol and adrenalin), and the release persists for at least 72 h [2].
- The acute release of noradrenaline from the sympathetic endings associated with the vascular endothelium results from tissue failure of the target organs of the disease. This release occurs in some specific diseases (severe pre-eclampsia, hypertensive retinopathy and renal crisis).
- There could be an exaggerated increase in the afterload (neurogenic pulmonary oedema, pheochromocytoma).
- Prior diseases might be present (myocardiopathy, coronaropathy, valvulopathy, atherosclerosis) depending on the age of the patients (young, elderly). The associated diseases preceding the hypertensive crisis, especially those of the basic organs that are essential for life and can trigger positive feedback, make the clinical picture. The occult, obvious or absent diseases define the signs and the symptoms related to a hypertensive crisis.

## Review

### Method

#### Literature search

The structure of the search strategy included a literature search of PubMed, MEDLINE, Cochrane Library and Google Scholar databases, as well as a review of the cited references by the identified studies and a hand search of relevant textbooks and reference works. The evaluated studies which have been identified are (1) terms to search for the health condition of interest: the diseases have been defined using explicit criteria for establishing their presence or not and (2) terms to search for the broad population and setting of interest evaluated: presence of a particular disease upon an hospital adult population (more than 18 years old).

English randomised control trials were searched for, which were published between January 1993 and February 2014.

#### Study selection

The following inclusion criteria were applied: prospective double-blind randomised controlled trials, experimental animal work studies, case–control studies and recruiting patients representative of the general sick population (i.e., adults over 18 years, diagnosed with the specific disease of interest). We did a general review of the literature without established hypothesis. The narrative review included analyses of 373 articles

### Neurogenic pulmonary oedema (NPE)

Neurogenic pulmonary oedema (NPE) is one clinical variation of hypertensive emergency. A high level of hypertension and extreme release of noradrenaline characterises it. Few reports on NPE have been written and it has not been well studied: an acute, protein-rich lung oedema occurring shortly after cerebral lesions associated with an acute rise in the intracranial pressure (traumatic head injury, haemorrhage or ischemia). Younger patients without any cardiopulmonary disease suffer from NPE. The age and sex of the patient are unimportant, and acute cerebral injury, endocranial hypertension and hyper-catecholaminaemia are the required events [3, 4]. If these steps do not occur, NPE does not develop.

The high level of norepinephrine causes hypertension, increased pressure in the left atrium and the pulmonary vasculature, electrocardiographic changes of ventricular repolarisation and the release of myocardial enzymes such as creatinine phosphokinase and troponin. The histologic examination of the myocardium shows patchy necrosis of the myocytes [5]. The seriousness of cerebral hypertension directly relates to the level of the catecholamines, and values over 2000 pg/ml are indicative of worse outcomes [3]. If an efficacy input on sympathetic endings or the failure of the baroreflex to trigger a negative feedback is observed, the cause has not been determined [6]. The type and severity of the endocranial hypertension or the participation of particular cerebral regions (points) likely play leading roles at the beginning of the disease. During NPE, the endocranial hypertension engages the hypothalamus and is associated with acute extrapyramidal dysfunction of the cerebellum and brainstem, which are the locations of the baroreflex and the chemoreflex, respectively. This might explain the defect of negative feedback and pressure regulation of hypertensive crises [7].

Cases of pheochromocytoma associated with pulmonary oedema show similar features [8–11].

### Cardiac acute pulmonary oedema with arterial hypertension

Cardiac acute pulmonary oedema is another clinical presentation of a hypertensive crisis. This condition occurs in older subjects with pre-existing, sometimes mild, cardiac disease.

The levels of catecholamines, norepinephrine and epinephrine, are higher during cardiogenic pulmonary oedema (CPE) compared with the values found in the same patients when they are not in an acute crisis [12].

The relationship between CPE and hypertension is a clinical and pathophysiologic link that explains the increase in the afterload and the storage of plasma upstream of the left cardiac chambers. The systolic function, normally preserved during CPE, is non-influential in the pathophysiology of CPE. The decrease in stroke volume, which better represents the diastolic function, is used to differentiate between symptoms or normality [13].

All of the types of acute hypertension, identified by the level and the duration, increase the afterload, placing increased pressure on the blood. The inability of acute dilation of the cardiac chambers causes the storage of blood in the left atrium and the pulmonary vascular network. If mitral failure is coincident, different effects develop.

### Hypertensive emergency during pregnancy

The appropriate terminology needs to be defined to discuss on *hypertensive crisis in pregnancy*.

Pre-eclampsia is classified as severe when a pregnancy that is more than 20 weeks of gestation is associated with arterial pressure greater than 160 and/or 90 mmHg and proteinuria greater than 1 g/24 h and there is organ injury (oliguria, cerebral injury, pulmonary oedema). The picture of HELLP syndrome (haemolysis, elevated liver enzymes and low-platelet count) that is sometimes associated with pre-eclampsia is rarely characterised by severe hypertension, which is not specific in its pathophysiology.

The cause of pre-eclampsia could be related to changes in the vascular tissue system of the uterus and placenta during pregnancy. The most likely hypothesis is that the placenta causes sensitising to catecholamine or allows soluble chemical substances to damage the vascular endothelium, such as the soluble Fms-like tyrosine kinase 1 (sFlt 1) and soluble endoglin. Increased sFlt 1 has been hypothesised to effectively reduce the concentration/activity of vascular endothelial growth factor, resulting in endothelial cell dysfunction, hypertension and proteinuria [14–16]. These data have been confirmed by experimental animal studies [17].

Reduced blood perfusion in all the organs develops because of severe vasoconstriction, which is a consequence of the increased sensitivity of the vascular network to pressurising chemical substances released from the uteroplacental system.

The mother’s organs suffer an acute blood supply reduction.

Regarding the renal blood flow, swelling of the glomerular endothelium has been shown documented, and the subendothelial deposits and detachment from the basement membrane lead to vascular obstruction [18]. These lesions are reversible in a time between 3 and 6 months post-partum and couple with the disappearance of hypertension. The vascular endothelium might be recognised as the target organ of pre-eclampsia [19] because of sensitised, hormonal hypertensive systems. The topic is not defined in the literature, and there are conflicting reports about the contributions of catecholamines vs. angiotensin and the characteristics of the subjects [20].

Pregnancy and pre-eclampsia are unique conditions that cannot be compared with non-pregnant subjects because the two conditions seem to bestow a heightened sensitivity to mediators. However, it seems that renin–angiotensin [14] and catecholamines might contribute to the conditions in humans [21, 22] and in animals [23].

Compared with hypertensive emergencies, the two systems, renin–angiotensin and catecholamines, though stimulated, are not as intensely increased as in neurogenic pulmonary oedema or hypertensive pulmonary oedema. For this reason, the target of their action appears to be sensitised. The role of other mediators, such as the endothelin system and vascular growth mediators, have not yet been defined [24, 25].

### Connective tissue diseases and scleroderma

Connective tissue diseases are a heterogeneous group of disorders that are associated with the production of autoantibodies. Subclinical or overt renal manifestations are frequently observed and complicate the clinical course of these illnesses.

Scleroderma is distinctive. Approximately 2–5 % of patients with scleroderma suffer from ‘renal crisis’, which is characterised by severe hypertension, rapidly progressive glomerulonephritis and crescent glomerulonephritis [26] with a decline in the renal function and thrombotic microangiopathy; this condition shows a significant benefit from early angiotensin-converting-enzyme inhibitor therapy and strict blood pressure control [27]. Renal crisis constitutes a rare and dangerous complication, and the affected patients present with prominent left heart failure and hypertensive encephalopathy [28]. Renal failure could be associated with moderate proteinuria without haematuria. Thrombotic microangiopathy is detected in 43 % of the cases.

The pathological anatomical framework shows a thrombotic microangiopathic process that particularly affects small vessels. Vascular changes are accompanied by thromboses, the accumulation of myxoid material and the development of onion-skin lesions and fibrointimal sclerosis later in the disease course [29]. Vascular damage is a primary event in the pathogenesis of scleroderma. The progressive vascular injury includes persistent endothelial cell activation/damage and apoptosis, intimal thickening, delamination, vessel narrowing and obliteration. These profound vascular changes lead to the vascular tone dysfunction and reduced capillary blood flow, with consequent tissue ischemia and severe clinical manifestations, such as digital ulcerations or amputations, pulmonary arterial hypertension and scleroderma renal crisis. The pathogenesis is thought to be a primary disease of the endothelium followed by a ‘vasculitis’ or ‘endotheliitis’. The release of specific mediators has a role in the onset of the ‘renal crisis’, and it is likely that endothelin and the renin–angiotensin system and aldosterone are involved [30].

### Hypertensive retinopathy and encephalopathy

In the literature, the two expressions ‘malignant hypertension’ and ‘accelerated hypertension’ should be considered conceptually equivalent [31]. Hypertensive retinopathy is characterised by a spectrum of retinal lesions associated with chronic and stable arterial hypertension (isolated microaneurysms, haemorrhages and cotton-wool spots, grade II–IV Keith–Wagener score). The clinical picture occurs in patients with certain characteristics: age greater than 40 years, chronic hypertension and cardiovascular risk factors, such as diabetes mellitus and dyslipidaemia. The condition is associated with multi-organ damage (renal failure, myocardial hypertrophy, atherosclerosis) and is predictive of stroke, congestive heart failure and cardiovascular mortality. The condition should be defined as *chronic hypertensive retinopathy*.

There is another clinical condition, *hypertensive retinopathy associated with acute hypertension*, which defines how malignant or accelerated the hypertension is in reference to organ damage and the speed of the disease appearance. This condition is rare and occurs in only 1 % of hypertensive patients [31]; it affects patients of younger ages. The affected individuals have with both primary and secondary hypertension, and it is more common in the black population of African origin.

The ophthalmoscopic examination shows the presence of bilateral retinal haemorrhages and exudates, with or without papilledema (grade IV Keith–Wagener score), and the fluoroangiographic examination shows retinal oedema [32]. This clinical picture is independent of cardiovascular risk and is seen in young males with the severe and abrupt appearance of arterial hypertension in patients with pheochromocytoma [32–34].

Because of the easy accessibility of funduscopic exams, the retinal changes play a paramount role in establishing the diagnosis, but these changes appear to be associated with obliterating endarteritis of other organs [35–37], particularly of the kidney, which is a common place of disease [38], as demonstrated by the reduction of filtration or by the presence of proteinuria.

Renal pathology is frequent, and renal endotheliitis is linked to accelerated hypertension. The acute or malignant or accelerated hypertension should be considered as a systemic disease with multiorgan involvement.

The causes of accelerated hypertension in a subject that had normal blood pressure or mild hypertension previously are not known. Studying the affected organs has not helped to determine the cause of the accelerated hypertension. The same histological lesions could be considered both as a cause and as an effect of acute hypertension. In the first case, an insult causing endothelial inflammation could lead to endotheliitis, which could lead to the release of pro-hypertensive factors with positive feedback on hypertension.

In the second case, the acute hypertension, of unknown cause, could be responsible for the endothelial injury.

Elevated blood pressure alone does not fully account for the extent of the retinopathy. If the acute rise in blood pressure appears to be the most likely cause of the endothelial lesion, the signs of endothelial injury are fibrinoid necrosis, oedema and endovascular cell proliferation.

The vascular endothelium serves as an important autocrine and paracrine organ and maintains vascular homeostasis by modulating the blood vessel tone and controlling homeostatic and inflammatory responses [39]. Endothelial dysfunction, which is suggested by the increase in inflammatory mediators, has been considered the onset of degenerative and proliferative and/or subsequent exudative lesions. From these observations, the concept of endotheliitis, a subacute inflammatory disease that could change the blood flow and cause hypertension, was developed.

This endotheliitis is vascular onion-skin associated with fibrinoid necrosis and arteriolar thrombosis involving other organs such as the kidney (renal failure and/or proteinuria) [31, 40], brain (posterior reversible encephalopathy) [41], gut or pancreas [35–38].

### Acute coronary ischemia

The natural history of acute ischemic coronary disease (angina and myocardial infarction) and dissecting aneurysms involves the appearance of pain.

Hypertension is a ‘life partner’ of ischemic cardiac diseases; it comes first and influences the prognosis and complications [42].

In the Western world, there is often a relationship between acute coronary syndrome and hypertension in 49–68 % of cases [43–45] and only 15 % of cases of age less than 30 years [46]. The link between intramural hematomas and a dissecting aneurysm with hypertension occurs in 100 % of cases [47].

Acute arterial hypertension, of new onset or associated with a pre-existing chronic hypertension, is present during episodes of myocardial ischemia, either transient ischemia (angina) or extended ischemia with the appearance of necrosis (myocardial infarction). The increased afterload and the wall stress worsen the basic disease.

The hypertensive crisis is a result of coronary heart disease, and there is pain. Hypertension triggers a positive feedback mechanism that results in myocardial ischemia. For this reason, a sharp reduction in blood pressure reduces the preload and the afterload and causes resolution or improvement in the pain of cardiac angina.

The type C myelinated sympathetic fibres carry the sensation of the pain. The stimulus to the nerve endings is produced by the release tissue and endothelial and platelet substances as a result of acute ischemia [48–50].

The pain represents an acute stressor that is adequate to cause an increase in blood pressure [49] through the release of substances such as adrenocorticotropic hormone and prolactin from the anterior pituitary gland, glucocorticoids from the adrenal cortex, epinephrine from the adrenal medulla and norepinephrine from the sympathetic nerves.

Any pain activates the sympathetic nervous system with a release of norepinephrine, as has been demonstrated in the clinic and experimentally [50–52]. There is no sufficient evidence showing that the onset of hypertension triggers the crisis of cardiac pain.

The *de novo* appearance of hypertension in cases of cardiac angina or ongoing aortic dissections is the result of ischemia, and the pain that occurs and the sympathetic nervous system and the activation of the pituitary-adrenal axis are intermediate mechanisms of disease.

### Stroke (intracerebral haemorrhage, subarachnoid haemorrhage, ischemic stroke)

The association of stroke with hypertension at the time of its presentation varies according to age in 55 to 100 % of the cases [53]. The appearance or worsening of pre-existing hypertension is part of the natural history of stroke. Within 72 h, the blood pressure values are reduced and return to the precrisis (precritical) levels.

The relationship between stroke and hypertension is significant, and the degree of control in acute stroke has been discussed [54].

The pathophysiological relationship between brain injury and the development of hypertension remains unclear. The acute stress response has a significant role. The extension of hypertension for up to 72 h after the acute stress proves this role [4]. Reactions to stressors typically involve either short- or long-term compensatory changes in the cardiovascular, endocrine, immune and somatosensory systems, which tend to maintain adequate physiological function against the imbalance created by the stressors [55]. Currently, there are not any elements to justify the existence of other pathogenic factors as causes of high blood pressure in stroke.

## Conclusion

‘Hypertensive emergencies’ are a group of diseases that are related to acute hypertension caused by catecholamines, the sympathetic nervous system, the vascular endothelium and acute stress. The hypertension is linked with the disease of one or more organs (myocardium, kidney, brain) in which the hypertension has a key role in the appearance of the pathology.

In clinical medicine, three novel concepts should be established: (a) ‘acute’ hypertension exists and is characterised by high arterial pressure values that could be targeted in a short time (in a few hours or a few days), (b) the acute hypertension is associated with organ disease and (c) this acute hypertension should quickly be treated because it causes more severe organ disease.

In ‘hypertensive urgency’, the stress has a low intensity, and the organs can endure this stress without being damaged.

Studies should explore why the regulation systems are temporarily absent and are not able to adjust for the hypertension.

### Proposal for new terminology

Having redefined the hypertensive emergency group of diseases that have acute arterial hypertension as a factor that could worsen the prognosis, we propose a classification scheme based on the hypertension pathogenesis:

- Acute primary hypertension or *reason* (*cause*) *of the disease*: a mechanism is established as the first moment of hypertensive disease targeting the heart, retina, brain and kidney. This category includes acute cardiac pulmonary oedema, neurogenic pulmonary oedema, pheochromocytoma, retinopathy and hypertensive encephalopathy acute glomerulonephritis and scleroderma.
- Acute secondary hypertension or *caused by disease*: a disease causes changes in the circulating volume or peripheral arteriolar resistance. This category includes acute glomerulonephritis, scleroderma and pre-eclampsia.
- Acute associated hypertension *without causal relationship*: the disease is the effect of an acute stress response. This category includes cardiac angina, acute myocardial infarction, dissecting aneurysm and stroke.

There could be different pathological mechanisms of the same disease, and different factors contribute to these different mechanisms. For this reason, the diseases of acute glomerulonephritis and scleroderma have been classified in both the primary and acute hypertension as secondary acute hypertension.
